# Incongruent visual cues affect the perception of Mandarin vowel but not tone

**DOI:** 10.3389/fpsyg.2022.971979

**Published:** 2023-01-04

**Authors:** Shanhu Hong, Rui Wang, Biao Zeng

**Affiliations:** ^1^Institute of Foreign Language and Tourism, Quanzhou Preschool Education College, Quanzhou, China; ^2^Department of Psychology, Bournemouth University, Poole, United Kingdom; ^3^School of Foreign Languages, Guangdong Pharmaceutical University, Guangzhou, China; ^4^EEG Lab, Department of Psychology, University of South Wales, Newport, United Kingdom

**Keywords:** incongruence effect, lexical tone, Mandarin, audiovisual speech, visual timing, lip movement, vowel

## Abstract

Over the recent few decades, a large number of audiovisual speech studies have been focusing on the visual cues of consonants and vowels but neglecting those relating to lexical tones. In this study, we investigate whether incongruent audiovisual information interfered with the perception of lexical tones. We found that, for both Chinese and English speakers, incongruence between auditory and visemic mouth shape (i.e., visual form information) significantly interfered with reaction time and reduced the identification accuracy of vowels. However, incongruent lip movements (i.e., visual timing information) did not interfere with the perception of auditory lexical tone. We conclude that, in contrast to vowel perception, auditory tone perception seems relatively impervious to visual congruence cues, at least under these restricted laboratory conditions. The salience of visual form and timing information is discussed based on this finding.

## 1. Introduction

Previous studies revealed that daily speech communication is bimodal, as listeners automatically integrate visual and auditory information during a face-to-face conversation (Massaro, 1987, 1998; Summerfield, 1987; van Wassenhove et al., 2005; Alsius et al., 2007; Valkenier et al., 2012; Marques et al., 2016). Due to the vast array of visual cues involved in audiovisual speech perception, many researchers have attempted to categorise them and evaluate their specific roles. First, these cues can be categorised by their location (i.e., head, face, and neck) as was the case for Wang et al. (2020). Second, from an acquisition perspective, Lalonde and Werner (2021) reviewed the audiovisual speech cues used by infants and children and highlighted the salience of temporal and phonetic information. The authors proposed a general perceptual, temporal mechanism that used primary visual cues such as onset and offset of speech and mouth movement. In addition to the general perceptual mechanism, the authors proposed a speech-specific phonetic/lexical mechanism that utilised salient visemic cues to perceive and recognise phonemes, syllables, and words. For instance, adults learn that a lip closure represents a bilabial sound, such as /p/, /b/, and /m/, and not a velar sound, such as /k/ or /g/. Third, cues may be organised according to their spatial and temporal features. Kim and Davis (2014) distinguished between visual form and visual timing information in speech perception. Visual form information is provided by the shape and movement of the mouth, lips, and tongue. On the contrary, visual timing information is derived from the perioral regions such as the head, neck, and eyebrows as well as from global facial movements.

Multiple visual cues, which originate from the face, head, and even body, are adopted and integrated into audiovisual speech perception. Among these visual cues, the most speech-specific cue is viseme. Chen and Massaro (2011) defined visemes as “units/categories of visual speech movements that were perceptually distinctive among the units/categories but much less so within each unit/category” (p. 956). Viseme is a well-established concept in audiovisual speech perception (Bernstein, 2012). It can be regarded as a good example and a precedent concept of “visual form” (Kim and Davis, 2014). Kim and Davis (2014) stated “that is, mouth and lip movements define shapes and spaces that can combine with tongue positions to provide form information about the identity of spoken segments. In addition, such motion provides timing information about segment onset, offset, and duration (Summerfield, 1979) and information about syllabic rhythmic structure from the cycle of jaw open-closure” (p. 86). In this study, we adopted viseme in the context of visual form and timing information and exemplified visual form with it.

Visemes are essentially units of speech that exhibit similar visual properties despite producing different sounds. While the visemic features of consonants are related to the manner and place of articulatory (e.g., bilabial plosive /b/, /p/; labio-dental fricative /f/, /v/), those of vowels are mainly related to the mouth shapes (e.g., the roundness of /o/ and flatness of /i/) involved in their production. Depending on what auditory stimuli are being perceived, some visual cues will be more important than others. Thus, visual cues may be organised into a salience hierarchy depending on their distinctiveness in a specific context. For instance, the place of articulation is a much more salient cue for bilabial consonants (e.g., /p/, /b/, or /m/) compared to velar consonants (e.g., /k/ or /g/). Developmental evidence also shows that some visual cues are acquired earlier than others. Infants and children learn to perceive and process noticeable visual cues earlier than other less noticeable cues; for instance, they may notice cues associated with a visually salient /b/ earlier than a less salient /g/ (Lalonde and Werner, 2021).

Previous studies assessing visual cues in spoken language tend to focus on segments of speech. However, lexical tones are typically suprasegmental in speech, which raises questions regarding whether any lexical tone-specific visual cues exist in audiovisual speech. According to Yip (2002, p.1), 60–70% of the world's languages are tonal. Like consonants and vowels, the lexical tone is a contrastive linguistic feature used to distinguish different meanings in spoken words, despite segments appearing identical. For example, there are four lexical tones in Mandarin Chinese, namely, /ma⌉/ (mā in Pinyin, Tone 1, high, 55, “mother”), /ma ↿/ (má, Tone 2, rising, 35, “hemp”), /ma ⇃⌉/ (mă, Tone 3, dipping, 214, “horse”), and /ma ⋎/ (mà, Tone 4, falling, 51, “scold”). Preliminary evidence (discrimination task: Burnham et al., 2001, 2015, 2022; identification task: Mixdorff et al., 2005; Burnham et al., 2006; Chen and Massaro, 2008) suggests that visual benefit occurs in lexical tone perception also, similar to that enhancing the discrimination and identification of consonants and vowels. Wang et al. (2020) categorised these visual cues into three types according to their origins, namely, head, eyebrows, and lip movements. However, as lexical tones are articulated by vibrations of the vocal cords, there are no apparent visemic cues from a speaker's mouth area. Thus, perhaps visemic cues involved in consonant and vowel perception are less relevant when it comes to lexical tone perception.

The focus on visual timing information (Kim and Davis, 2014) sheds light on the way visual cues might facilitate lexical tone perception. Visual form information is less relevant in this study, as lexical tones do not provide distinct form cues, e.g., mouth shape and articulatory manner. Although lexical tones lack salient visual form cues, it is well-known that lexical tone perception is dominated by F0 and is attributed to other acoustic features, e.g., amplitude and duration. Among Mandarin lexical tones, the dipping tone is the longest in acoustic duration, and it is significantly longer than the shortest falling tone (Xu, 1997; Attina et al., 2010; Reid et al., 2015). Moreover, Attina et al. (2010) showed that non-rigid face movement could explain 95.49% of the tonal variances for words spoken in a citation format. The principal component analysis method identified six principal components (PCs) and revealed the two most powerful PCs, namely, jaw opening and lip protrusion, which explained 66.85 and 17.99% of the variance, respectively. Although it is difficult to identify specific visual form cues that might aid lexical tone perception, for the aforementioned reasons, visual timing cues such as lip movement duration, might be more relevant, especially when comparing Tone 3 and Tone 4, which have the greatest difference in duration between any tone pairings. Therefore, given the differences in duration, we argue that lip movement duration might be one visual cue that listeners can use to distinguish between tones. We argued that the acoustic duration of each tone might be mapped to the duration of lip movements.

First, auditory tone duration is systematically related to visual cue duration. A longer auditory tone has a longer visual cue duration. In our case, Tone 3 has a long auditory and visual duration, whereas Tone 4 has a shorter auditory and visual duration. Second, some audiovisual prosodic studies found that prosodic visual cues are located in the mouth regions (e.g., lips, chin, and jaw), and these cues might be related to the intensity or duration of spoken words since stress syllables/words with greater intensities result in faster lip and jaw movements (Krahmer and Swerts, 2001; Scarborough et al., 2009).

In this study, we propose that lip movement duration might be a potential visual timing cue facilitating audiovisual lexical tone perception. For instance, an acoustically long Tone 3 relies on prolonged lip movements, and these movements might be integrated into the audiovisual processing of lexical tone and might help listeners identify lexical tones in the same way visual form information aids identification of consonants and vowels (Xie et al., 2018). Many studies have revealed that the integration of congruent auditory and visual information can facilitate speech perception and shorten response time in identification tasks, while incongruent information slows down response time and reduces identification accuracy, known as the incongruence effect (Robinson and Sloutsky, 2007; Baart et al., 2014; Irwin and DiBlasi, 2017). The incongruence effect can be used to investigate whether lexical tone perception is hindered by mismatched auditory and visual information, specifically mismatched lip movement duration.

Furthermore, many studies showed that the listener's native language could affect the impact of visual information on speech perception (Sekiyama and Tohkura, 1991, 1993; Sekiyama, 1997; Hazan et al., 2010; Magnotti et al., 2015). Vowels are a universal speech component for both Chinese and English speakers, and thus, for vowel perception, both sets of participants will demonstrate an incongruence effect when the visual information is inconsistent with the auditory information. In terms of lexical tone perception, many studies have revealed that non-native Chinese speakers, such as English or Dutch speakers, adopt universal perception strategies that allow them to utilise some visual cues (Burnham et al., 2015; Han et al., 2019). Wang et al. (2020) noted that native tonal language speakers outperformed non-native tonal language speakers in an audiovisual condition; however, the non-natives showed superior performance in some cases of the visual-only condition (Smith and Burnham, 2012; Burnham et al., 2015). Burnham et al. suggested that non-tonal language speakers explored facial cues as they found themselves in a challenging phonetic situation. However, the non-native speakers' superior performance in the visual-only condition did not necessarily transfer to the audiovisual condition.

As few cross-language studies (Hannah et al., 2017) have been concerned with audiovisual incongruence effects on lexical tones, we intend to address this gap by further exploring whether differences exist between Mandarin and English speakers processing Mandarin lexical tones. Specifically, we will manipulate lip movement duration, which is proposed to be a visual timing cue for lexical tones, and investigate whether it induces an incongruence effect on lexical tone perception. For vowel perception, we attempt to produce an incongruence effect by manipulating mouth shapes, as they represent a key visual form cue. To encourage the listener to make use of visual cues, background noise will be embedded into the experiment (Mattys et al., 2012). Babble noise was chosen for the background noise as it masks information better than other noise, e.g., white noise (Mixdorff et al., 2005, 2006).

To sum up, three main research hypotheses were addressed:

1. *The incongruence effect*: This effect is characterised by reduced discriminability of auditory information and delayed response times. For lexical tone perception, we predict that an incongruence effect will emerge when lip movement duration does not match the auditory tone, for instance, a combination of auditory /à/ (Tone 4) and visual /ă/ (Tone 3). For vowel perception, we predict that a similar incongruence effect will occur when the mouth shape is inconsistent with the auditory vowel, for example, a combination of auditory /a/ (requiring a round mouth shape) and visual /i/ (displaying a flat mouth shape).
2. *The interaction between the incongruence effect and the listening conditions*: It is predicted that there will be a significant interaction between the incongruence effect and the listening conditions. More specifically, the incongruence effect will be stronger in the noisy condition compared to the clear condition.
3. *The interaction between the incongruence effect and language*: For the lexical tone task, we hypothesise that lip movement duration will be more salient for Chinese than English speakers in the audiovisual mode, and thus, we predict that Chinese speakers will show a larger incongruence effect than English speakers. Alternatively, the incongruence effect for lexical tone perception may only occur in Chinese speakers.

## 2. Materials and methods

### 2.1. Design

This study consists of a tone identification and a vowel identification task. Both tasks employed a 3-way 2 × 2 × 2 mixed design with 2 levels of language (Mandarin and English) × 2 levels of incongruence (congruent and incongruent) × 2 listening conditions (clear and noisy). The language was the between-subject factor, while listening conditions and incongruence were the two within-subject factors. For each experiment, three factors were manipulated: language—participants were native Mandarin or English speakers; incongruence—the video displayed congruent or incongruent audiovisual information; and listening condition—participants watched the video under clear or noisy conditions. For example, in the tone identification experiment, a congruent stimulus used the auditory stimulus /a/ produced with the dipping Tone 3 and dubbed with the visual stimulus /a/ produced with the same Tone 3 (A/ă/V/ă/). In the incongruent condition, the audiovisual combination A/ă/V/à/ incorporated an auditory /a/ stimulus, which was produced with the dipping Tone 3, dubbed to the visual /a/ stimulus, which was produced with the falling Tone 4, or *vice versa*. Similarly, in the vowel identification task, the congruent condition was referred to as A/ă/V/ă/, which involved an auditory /a/ dubbed to a visual /a/ where both stimuli were presented in Tone 3. However, in the incongruent condition, A/ă/V/ĭ/ was a combination of an auditory /a/ stimulus, which was presented in Tone 3, dubbed to a visual /ĭ/ stimulus, which was also presented in Tone 3, or *vice versa*.

### 2.2. Participants

A total of 21 native Mandarin speakers from Mandarin-speaking areas, born in China, and educated at universities in China (13 women; 26.7 ± 6.1 years), and 32 monolingual native English speakers from Bournemouth University, UK (17 women; 19.2 ±1.0 years), were tested in both tasks (n=53). All Mandarin and English participants reported normal or corrected-to-normal visual acuity, and none reported previous hearing impairments. All participants gave written informed consent to participate in the experimental protocol approved by the Institutional Review Board of Bournemouth University.

### 2.3. Materials

The raw stimuli were three Mandarin audiovisual monosyllables; ă, à, and ĭ. ă is a combination of a round mouth-shaped vowel and dipping Tone 3 (T3), à is a combination of a round mouth-shaped vowel and falling Tone 4 (T4), while i is a combination of a flat mouth-shaped vowel and dipping Tone 3. Two vowels were used in the vowel identification task (round /a/ and flat /i/), and two lexical tones were used in the tone identification task (long /ă/ and short /à/). Each monosyllable was produced in two tokens by one male native Mandarin speaker (aged 24 years) in order to avoid audiovisual feature-specific processing or low-level processing, in which participants may pay more attention to specific features of the audiovisual syllables such as their acoustic differences or any mouth-movement differences, rather than focusing on the phonetic features of the syllables. The raw videos were recorded using a Nikon D3300 camera, and the auditory materials were recorded using Audacity (Copyright 2021 Audacity Team). The video recordings were edited using Adobe Premiere Pro CC (Adobe Systems, California), into clips with 1,280 × 720 resolution and a standardised rate of 29.97 frames per second (1 frame = 33.37 ms). In the video, only the lower half of the speaker's face was presented (eyes were not shown). The soundtracks (48 kHz with 32-bit amplitude and 9 dB SNR) of the videos were edited in Adobe Audition CC (Adobe Systems, California). The SNR-9dB babble noise (Mixdorff et al., 2005) contained mixed voices of news storey readings in Mandarin produced by six Mandarin native speakers (three women and three men). All experimental materials are depicted in Table 1.

**Table 1:** The duration of the audiovisual stimuli, corresponding to auditory, and visual components and onset times (in ms).

**Task**	**Congruence**	**Syllable**	**Lip onset**	**Audio onset**	**Lip movement duration**	**Audio duration**
Tone	Congruent	A/ă/V/ă/	267	767	1,535	830
	Congruent	A/à/V/à/	250	767	1,268	480
	Incongruent	A/ă/V/à/	250	767	1,268	830
	Incongruent	A/à/V/ă/	267	767	1,535	480
Vowel	Congruent	A/ă/V/ă/	267	767	1,535	830
	Congruent	A/ĭ/V/ĭ/	684	767	1,034	903
	Incongruent	A/ă/V/ĭ/	684	767	1,034	830
	Incongruent	A/ĭ/V/ă/	267	767	1,535	903

**Figure 1:**
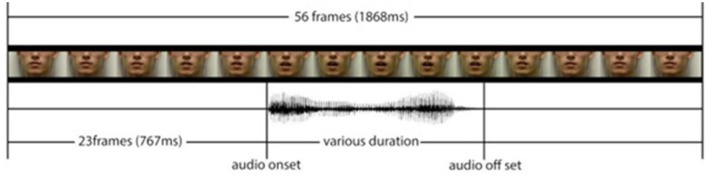
Trial structure in both tasks.

The duration of the whole audiovisual stimulus presentation was 1,868 ms, in which the gap between the visual stimulus onset and the auditory onset was 767 ms (see Figure 1). The sound was always released at the 767 ms time point, and the lip movement duration, which was calculated as the length of time between the opening and closing of the mouth, was always longer than the auditory stimulus. This is because, in natural speech, lip movements always occur before the sound is produced (~100 ms before).

Each experiment contained 300 trials presented in six blocks, 50 trials per block. Two buffer trials were presented at the beginning of each block, 12 in total. The buffer trials were randomly selected from the experimental trials in the clear condition and were not included in the final data analysis. Thus, the total number of trials for each experiment was 288. They consisted of 2 tones (/ă/ and /à/) or 2 vowels (/a/ and /i/) × 2 tokens × 2 listening conditions (clear and noisy) × 2 levels of congruence (congruent and incongruent) × 18 repetitions. Each block contained 48 trials made up of random combinations of the independent variable conditions (i.e., vowels or tones presented to Mandarin or English speakers, under clear or noisy conditions with congruent or incongruent visual information).

### 2.4. Procedure

Both tasks were conducted in a sound-attenuated chamber, with participants facing a 21-inch LCD monitor. The sounds were played through Sennheiser D 280 headphones, at a volume of ~65 dB SPL. Participants were seated in front of the computer monitor in a comfortable chair with headphones on. The order of presentation was shuffled between participants using E-prime 2.0. Participants were given opportunities to take short breaks between each of the six blocks. A two-alternative forced choice paradigm (2AFC) was applied in this study. During this session, participants were presented with a fixation cross (“+”) in the middle of the monitor which lasted for 1,000 ms, followed by a syllable in a video clip.

The participants were expected to react to the given syllable as accurately and quickly as possible. They were required to listen attentively and watch the articulating mouth in the video clips while indicating what they had heard by pressing a key. In the tone identification task, the participants watched the audiovisual video clips and responded to the auditory syllable /a/ in Tone 3 by pressing the corresponding key “P” on the keyboard and to the auditory /a/ in Tone 4 by pressing the corresponding key “Q.” In the vowel identification task, the participants responded to the auditory syllable /a/ by pressing the corresponding key “P” on the keyboard and to the auditory syllable /i/ by pressing the corresponding key “Q.”

To familiarise themselves with the procedure, both the Chinese and the English participants undertook a short practise session before the formal test began. Given that the English participants had no linguistic experience in Mandarin and that they lacked basic phonetic knowledge of a tonal language, they were provided with a brief training session prior to the tone identification task. They were told that there are four tones in Mandarin and were asked to imitate the experimenter producing the four tones of /a/. Participants then performed an identification test under clear conditions and were required to score above 75% response accuracy to progress to the formal test.

### 2.5. Data analysis strategies

A three-way repeated measures ANOVA was conducted to analyse the reaction time (RT) and *d* prime (*d*′). RT was calculated from the onset of the audiovisual stimulus and log-transformed in the analysis. The *d*′ calculator (Gaetano et al., 2015) was adopted to measure sensitivity rather than accuracy rate, as the latter is affected by both sensitivity and bias. *d*′ is a measure of an individual's ability to detect signals.

To illustrate how a single participant's *d*′ was calculated in the lexical tone task, an example is provided. When the presented lexical tone was Tone 3 or Tone 4 in a congruent and clear listening condition, one Chinese participant responded by judging what lexical tone they had heard. Over 72 trials [for instance, 2 tones (/ă/ and /à/) × 2 tokens × 18 repetitions], half the tones are Tone 3, and half the tones are Tone 4. We defined Tone 3 as the signal and Tone 4 as the noise. There are four possible outcomes for a participant. For example: (1) *Hit*: The target was Tone 3, which was judged correctly as Tone 3, response frequency: 34. (2) *Miss*: The target was Tone 3, which was judged wrongly as Tone 4, response frequency: 2. (3) *False Alarm*: The target was Tone 4, which was judged wrongly as Tone 3, response frequency: 0. (4) *Correct Rejection*: The target was Tone 4, which was judged correctly as Tone 4, response frequency: 36. The Chinese participants' *d*′ is calculated using their response frequencies under specific conditions (e.g., congruent and clear listening conditions), by subtracting the standardised false alarm frequency from the standardised hit frequency. The calculator uses response frequencies from a single participant under a set of specific conditions. Thus, a total of 8 *d'* scores will be created for all combinations of the three factors (listening conditions, language, and congruency). Similarly, the *d*′ calculator was also applied in the vowel identification task. We defined /ă/ as the signal and /ĭ/ as the noise. The other calculations are identical to those in the lexical tone condition.

## 3. Results

### 3.1. Lexical tones

Analysis of the *d'* values showed that the main effect of the listening condition was significant: *F*_(1,51)_ = 74.81, *p* < 0.001, $\eta_{\text{p}}^{2}$ = 0.60, which indicated that the listeners discriminated between the two tones better in the clear condition (*mean* = 3.81, *SE* = 0.11) than in the noisy condition (*mean* = 2.87, *SE* = 0.11). The main effect of language was also significant: *F*_(1,51)_ = 17.14, *p* < 0.001, $\eta_{\text{p}}^{2}$ = 0.25, which indicated that in all conditions the Mandarin speakers (*mean* = 3.77, *SE* = 0.16) discriminated between the two tones better than the English speakers did (*mean* = 2.91, *SE* = 0.13). The main effect of incongruence was not significant (*p* = 0.06), demonstrating that incongruent audiovisual information did not interfere with the identification of lexical tone. The interactions of these factors were not significant (*ps* > 0.51; see Table 2).

**Table 2:** The mean *d'* and RT (in ms) of the lexical tone task for Mandarin (*N* = 21) and English speakers (*N* = 32).

	**Condition**	**Mandarin**				**English**			
		**Congruence**		**Incongruence**		**Congruence**		**Incongruence**	
		**Mean**	**SD**	**Mean**	**SD**	**Mean**	**SD**	**Mean**	**SD**
*d′*	Clear	4.34	0.79	4.14	0.72	3.48	0.89	3.46	0.99
	Noisy	3.31	0.77	3.26	0.74	2.39	0.93	2.32	1.11
RT	Clear	1,277	57	1,275	60	1,326	108	1,332	110
	Noisy	1,389	98	1,387	103	1,401	133	1,392	124

Analysis of RT showed that only the main effect of the listening condition was significant: *F*_(1,51)_ = 70.15, *p* < 0.001, $\eta_{\text{p}}^{2}$ = 0.58. The two-way interaction between the listening condition and language group was also significant: *F*_(1,51)_ = 4.68, *p* = 0.035, $\eta_{\text{p}}^{2}$ = 0.08. Other interactions failed to reach significance (*ps* > 0.16). A pairwise comparison of the language group and listening condition interaction demonstrated that the RT of Mandarin speakers (mean = 1,276 ms, SE = 20) was significantly shorter than that of English speakers (mean = 1,329 ms, SE=16) in the clear condition (*p* = 0.045). However, there was no significant difference in RT between the Mandarin and English speakers in the noisy condition (*p* = 0.78; see Table 2).

### 3.2. Vowels

The same ANOVA was applied to the RT and *d*′ for vowels (see Table 3). The results from the *d'* analysis showed that the main effect of incongruence was significant: *F*_(1,51)_ = 93.88, *p* < 0.001, $\eta_{\text{p}}^{2}$ = 0.65, which indicated that the listeners discriminated between the two tones better in the congruent condition (*mean* = 4.08, *SE* = 0.12) than in the incongruent condition (mean = 2.77, SE = 0.15). The main effect of the listening condition was also significant: *F*_(1,51)_ = 68.59, *p* < 0.001, $\eta_{\text{p}}^{2}$ = 0.58, which indicated that the listeners discriminated between the two tones better in the clear condition (*mean* = 3.84, *SE* = 0.13) than in the noisy condition (*mean* = 3.01, *SE* = 0.13). However, the main effect of the language group was not significant (*p* = 0.71). The two-way interaction between listening condition and incongruence was significant: *F*_(1,51)_ = 48.09, *p* < 0.001, $\eta_{\text{p}}^{2}$ = 0.49. A further three-way interaction of listening condition, congruence, and language was also significant: *F*_(1,51)_ = 5.46, *p* = 0.02, $\eta_{\text{p}}^{2}$ = 0.10. To further probe the simple effect of the three-way interaction, the difference in *d*′ between congruent and incongruent conditions was used to compute an effect size for the incongruence effect in both clear and noisy listening conditions. Pairwise comparisons showed the effect size of the incongruence effect was significant in both clear and noisy conditions (*p*s < 0.001). However, an independent sample *t*-test was run to compare differences in effect sizes between clear and noisy conditions amongst Chinese and English speakers. The results showed that, in the noisy listening condition only, the effect size of Chinese speakers (*mean* = 2.42, *SE* = 0.44) was significantly larger than that of English speakers (*mean* = 1.66, SE = 0.20): *t*_(1,51)_ = 1.76, *p* < 0.05, but there was no significant difference in effect size between Chinese and English speakers in the clear listening condition.

**Table 3:** The mean *d'* and RT (in ms) of the vowel task for Mandarin (*N* = 21) and English speakers (*N* = 32).

	**Condition**	**Mandarin**				**English**			
		**Congruence**		**Incongruence**		**Congruence**		**Incongruence**	
		**Mean**	**SD**	**Mean**	**SD**	**Mean**	**SD**	**Mean**	**SD**
*d′*	Clear	4.21	1.16	3.74	1.15	4.06	0.70	3.37	0.97
	Noisy	4.17	1.18	1.75	1.77	3.89	0.78	2.22	1.10
RT	Clear	1,272	88	1,325	86	1,255	100	1,306	108
	Noisy	1,304	75	1,418	89	1,308	91	1,399	100

Analysis of RT for vowels showed that the main effect of incongruence was significant: *F*_(1,51)_ = 267.04, *p* < 0.001, $\eta_{\text{p}}^{2}$ = 0.84. The main effect of listening condition was significant: *F*_(1,51)_ = 107.54, *p* < 0.001, η^2^_*p*_ = 0.68. However, the main effect of language group was not significant (*p* = 0.56). A two-way interaction between listening condition and incongruence was significant: *F*_(1,51)_ = 38.63, *p* < 0.001, $\eta_{\text{p}}^{2}$ = 0.43. No other interactions reached significance (*ps* > 0.18). Pairwise comparisons involving incongruence and listening condition showed that the RT of the incongruent vowels was significantly longer than the RT of the congruent vowels in both clear (mean = 1,315 ms, SE=14, vs. mean = 1,263 ms, SE = 13, *p* < 0.001) and noisy (mean = 1,409 ms, SE = 13 vs. mean = 1,306 ms, SE = 12, *p* < 0.001) conditions (see Table 3). To further probe this interaction between incongruence and listening conditions, the difference between congruent and incongruent RT in clear and noise conditions was compared using a paired sample *t*-test, and the result showed that the effect size of the incongruence effect in the noisy condition (mean = 100 ms, SE = 7) was significantly larger than the effect size in the clear condition (mean = 52 ms, SE = 5): *t*_(1,52)_ = 6.31, *p* < 0.001.

## 4. Discussion

The results demonstrated that the perception of lexical tones was not altered by the incongruence between auditory and visual timing information, specifically lip movement duration. On the contrary, for vowel perception, incongruent visual form information (i.e., mouth shape) delayed the response time and decreased speech discriminability. Both English and Mandarin participants were influenced by visual form information under noisy listening conditions. The current finding is consistent with Han et al. (2020), who found that visually salient lip-reading-based cues aided consonant perception but failed to find any positive impact of head and neck movement cues for Mandarin lexical tone perception. In addition, although we assumed that vowels were a universal feature for both Chinese and English speakers, the RT results demonstrated that, in the noisy condition, Chinese listeners experienced a greater incongruence effect than English listeners, which indicates that Chinese listeners might be more sensitive to their native language. We suggest that this might be caused by a “native language effect” that has been reported in previous studies (Sekiyama and Tohkura, 1993; Wang et al., 2009).

Visual timing cues have rarely been studied in the field of lexical tone perception, although the distinction between visual temporal/timing cues and phonetic/form cues has been raised in recent years (Kim and Davis, 2014; Lalonde and Werner, 2021). Lalonde and Werner (2021) developed the argument of visual temporal properties in audiovisual speech and attributed them to a general perceptual, temporal mechanism. This mechanism can induce temporal expectancy and accelerate speech detection and recognition, as visible mouth movements precede acoustic speech by 100–300 ms (Chandrasekaran et al., 2009).

However, this study differs from the approach of temporal expectancy and postulated that lip movement duration was one speech-specific cue rather than a non-specific perceptual feature. The lip movement duration was mapped to the acoustic lengths of two contrasting Mandarin tones, namely, the longest dipping tone (visual: 1,535 ms vs. auditory: 830 ms) and the shortest falling tone (visual: 1,268 ms vs. auditory 480). Indeed, previous work suggested that lip movement duration could help listeners distinguish between Tone 3 and Tone 4 (Xie et al., 2018). However, the current incongruence results suggest that visual timing information is a less salient cue compared to visual form information for vowel perception (e.g., mouth shapes).

The lack of incongruence effect may lead to questions about the salience of visual cues for lexical tones. Lalonde and Werner (2021) suggested that visual phonetic and temporal cues were organised hierarchically, by referring to their distinctiveness and order of acquisition. Developmental evidence (Lalonde and Holt, 2015; Weatherhead and White, 2017) indicated that infants and children acquired basic temporal cues, e.g., the onset of speech and visually distinct phonetic cues such as visemic cues earlier than other subtle speech-specific visual cues. Initially, the salience hierarchy was based on studies of segmental consonants and vowels. These studies emphasised visual form and did not take visual timing or temporal cues into account. Furthermore, visual timing cues (e.g., segment onset, offset and duration, and information about syllabic rhythmic structure from the jaw opening and closing cycle) have been proposed and investigated by Kim and Davis (2014). However, lip movement duration, which was the timing cue in this study, was not included in the visual timing categories. Therefore, lip movement duration might represent a subtle temporal cue that is less salient than other basic temporal cues such as the onset and offset of lip movements.

Lip movement duration is one of the most understudied cues, although it may still be relevant to lexical tone perception. Multiple cues have been proposed over the last two decades, including mouth, face, neck, and head movements. However, these cues can be regarded as a combination of form and timing information, and thus, it is difficult to determine their specific mechanisms of action that aid lexical tone perception. Even a single motion, observed in the face or head, could be analysed in spatial (form) and temporal (timing) dimensions to determine which is more salient. Thus, perhaps it is time to take salience into account when examining the contribution of specific visual cues for lexical tone perception. This could be beneficial for a number of reasons.

First, these visual cues might relatively contribute to a specific task or target. For instance, Burnham et al. (2022) showed that tone discrimination involved both face and head motion, but for phoneme (consonants and vowels) discrimination, face motion was sufficient and the head motion was irrelevant. Second, these visual cues are defined by their origins, such as from the lip, face, or head, rather than their functions. Salience analysis could evaluate their relative importance and configuration in audiovisual speech perception. The distinction between form and timing has framed how general and speech-specific information is perceived and integrated into audiovisual perception and recognition.

In addition, focusing on the salience of visual cues leads us to another important question regarding what information is pertinent when visual form and timing cues coexist. For instance, in the present vowel perception task, the visual timing and form information might be confounded. The stimulus incorporated an auditory A/ă/ and a visual V/ĭ/. The original lip movement duration of /ă/ was 1,535 ms, whereas the lip movement duration of /ĭ/ was relatively short: 1,034 ms. This difference of 501 ms is even larger than the difference between Tone 3 /ă/ and Tone 4 /à/, which was 267 ms, and might have contributed to the listener's ability to correctly identify the vowels. Another potentially confounding factor is the onset of lip movement, as the lip movements of /ĭ/ started at 684 ms; 417 ms later than the onset of lip movements for /ă/, which began at 207 ms.

Consequently, we found an incongruence effect for vowel perception, but could not determine whether this was a result of mouth shape or lip onset/lip movement duration. While visual form information, such as unmatched mouth shape (for instance, A/ă/V/ĭ/), might delay response time and distort the perception of the auditory vowel /a/, it is possible that visual timing information, such as lip onset time (compared to congruent A/ă/V/ă/ at 267 ms, the incongruent A/ă/V/ĭ/ starts at 684 ms) or lip movement duration (compared to the congruent A/ă/V/ă/ which has a duration of 1,535 ms, the incongruent A/ă/V/ĭ lasts only 1,034 ms), may also be a contributor. Future research should look to differentiate the roles of form and timing information and examine their relative contributions.

Future research should also look to employ more sensitive measures. For instance, the incongruence effect was measured using the 2AFC task, which merely indicated whether Chinese and English speakers were confused by the mismatched auditory and visual information, and it does not reveal what the listeners actually perceived. Previous studies have demonstrated that a McGurk effect can occur for incongruent audiovisual vowel perception (Traunmüller and Öhrström, 2007; Valkenier et al., 2012); thus, future studies may wish to adopt the McGurk paradigm to investigate what percept occurs during the audiovisual processing of incongruent lexical tones.

Overall, visual timing information has received less attention in previous studies and still requires more work to understand its role in audiovisual speech perception. The present findings suggest that form information was a salient cue for vowel perception, but for both Chinese and English speakers, visual timing information in the form of lip movement duration was not as salient for lexical tone perception.

## Data availability statement

The raw data supporting the conclusions of this article will be made available by the authors, without undue reservation.

## Ethics statement

The studies involving human participants were reviewed and approved by Bournemouth University. The patients/participants provided their written informed consent to participate in this study.

## Author contributions

BZ developed the research idea, was actively involved in the data analysis, coordinated the co-authors in writing sections of the manuscript, wrote paragraphs along with the manuscript, and edited the final work. SH co-developed the research idea, performed data collection, recruited participants, co-authored the introduction, results, discussion sections, and contributed to the organisation of the manuscript. RW analysed the data, authored the results section under the guidance of BZ and collaborated with SH in planning and running additional statistical analyses that were necessary for this study. All authors contributed to the article and approved the submitted version.
